# Perspectives on sexual history taking in routine primary care consultations in North West, South Africa: Disconnect between patients and doctors

**DOI:** 10.4102/phcfm.v14i1.3286

**Published:** 2022-06-09

**Authors:** Deidré Pretorius, Motlatso G. Mlambo, Ian D. Couper

**Affiliations:** 1 Division of Family Medicine, Faculty of Health Sciences, University of the Witwatersrand, Johannesburg, South Africa; 2 Institutional Research, Department of Research Intelligence, University of South Africa, Pretoria, South Africa; 3 Ukwanda Centre for Rural Health, Faculty of Medicine and Health Sciences, Stellenbosch University, Cape Town, South Africa

**Keywords:** barriers, sexual history taking, receptiveness, patient–doctor engagement, communication, patient-centredness, sexual dysfunction

## Abstract

**Background:**

Sexual history is rarely taken in routine consultations and research reported on common barriers that doctors experience, such as gender, age and cultural differences. This article focuses on how patients and doctors view sexual history taking during a consultation and their perspectives on barriers to and facilitators of sexual history taking.

**Aim:**

This study aimed to explore doctors’ and patients’ perspectives on sexual history taking during routine primary care consultations with patients at risk of sexual dysfunction.

**Setting:**

The research was conducted in primary care facilities in the Dr Kenneth Kaunda Health District, North West province.

**Methods:**

This was part of grounded theory research, involving 151 adult patients living with hypertension and diabetes and 21 doctors they consulted. Following recording of routine consultations, open-ended questions on the demographic questionnaire and brief interactions with patients and doctors were documented and analysed using open inductive coding. The code matrix and relations browsers in MaxQDA software were used.

**Results:**

There was a disconnect between patients and doctors regarding their expectations on initiating the discussion on sexual challenges and relational and clinical priorities in the consultation. Patients wanted a doctor who listens. Doctors wanted patients to tell them about sexual dysfunction. Other minor barriers included gender, age and cultural differences and time constraints.

**Conclusion:**

A disconnect between patients and doctors caused by the doctors’ perceived clinical priorities and screening expectations inhibited sexual history taking in a routine consultation in primary care.

## Introduction

Doctors’ consultation skills in relation to patients’ sexual health in primary healthcare settings are largely unreported in South Africa. The doctor and patient engage around disease and medication during the clinical consultation in order to improve patient health.^1^ Both bring their own past life, disease and illness experiences, as well as cultural and gender perceptions, to the consultation. These attitudes and perceptions can act as barriers in the discussion and management of sexual challenges or can facilitate the sharing of sexual history by the patient. If no screening regarding sexual health occurs during a consultation, patients may leave feeling their expectations were not met or even negated.^2^ Not addressing sexual challenges amongst patients may increase their sexual risk-taking to compensate for perceived sexual shortcomings, which may again lead to sexually transmitted diseases and even relational conflict or violence.^3,4^

Some researchers suggest that spontaneous disclosure of sexual dysfunction occurs in approximately 3.0% of all consultations, irrespective of the diagnosis of the patient, which can improve to 16.0% if doctors ask about it, whilst others postulate it to vary between 14.0% and 20.0%.^5,6,7,8^ Most of the studies on barriers of sexual history taking refer to high-risk patients, such as patients living with cardiovascular disease, hypertension, diabetes and other chronic conditions; these patients often form a proportion of all the participants. In one of the biggest primary care retrospective studies of general patient visits in the Bronx, United States (US), (*n* = 1347) researchers found that there was no history taking documented in 65.0% of the visits and a full sexual history was taken in only 1.8% of the visits.^9^ Doctors are adamant that they will screen for sexual dysfunction when it is clinically indicated, by which they mean if it is the presenting complaint.^10,11^ A mail survey conducted in a primary care setting in Atlanta, Georgia, documented high rates of self-reported screening; 58.0% of physicians in four specialties (obstetrics and/or gynaecology, internal medicine, general and/or family practice, and paediatrics) claimed to ask their patients about sexual activity routinely, which improved to 79.0% if it was clinically indicated and relevant to the chief complaint.^12^ Conversely, another US study found that nearly 77.0% of geriatric fellows and programme directors were reluctant to take a sexual history in elderly patients and none were screened for sexually transmitted infections because of gender and age differences and general discomfort with the topic.^13^ About 30.0% of doctors in Ireland considered themselves comfortable to discuss sexual topics with patients^14^ Research on sexual dysfunction in South Africa is rare. A systematic review that covered research on sexual dysfunction, drawing on a South African sample between 1970 and 2014, reported no research on sexual history taking.^15^

Sexual history taking research usually reflects on perceptions of and barriers to sexual history taking. Common barriers are gender discordance, age differences between patient and service provider, sexuality in older people, culture, sexual orientation and poor communication.^2,7,11,16,17,18,19,20,21^ These studies suggested that these barriers led to patient discomfort and fear of dismissal of their complaints. In Toronto, nearly 64% of female patients under the age of 30 years and 50% of nulliparous patients considered a male doctor or medical student taking a sexual history as problematic.^22^ It seems that gender discordance in sexual history taking may be more than just dealing with the perceived gender of the patient or professional. Research suggested that female doctors, compared with their male counterparts, were perceived to be more empathic and did not interrupt the patient during the consultation, which facilitated disclosure of sexual challenges.^22^ Another study in the US highlighted that more system-related barriers, such as time constraints, no effective management, the number of patients to consult, years in practice or practice experience, were significant issues that should be overcome to treat sexual dysfunction.^21^ In-depth literature search revealed significant dearth of information on sexual history taking for sexual dysfunction in sub-Saharan countries.

Considering the worldwide low screening rate and numerous barriers to sexual history taking, it seems healthcare workers do not perceive sexual dysfunction to be a big problem. Globally, however, up to 31% of men and 39% of women live with sexual dysfunction^23,24^ and this percentage increases significantly if certain medications and diseases are present^25,26,27,28^ Considering erectile dysfunction as a biomarker for coronary artery disease, it must be a screening priority.^29,30,31^ Although sexual health research is not common in South Africa, research on male sexual dysfunction has raised serious red flags about the proportion of patients living with sexual challenges.^31,32,33^ The most recent South African study found that 97% of diabetic male patients who frequented a primary healthcare clinic lived with erectile dysfunction.^33^ In addition, in South Africa, it has been reported that sexual challenges often result in conflict in intimate relationships, increase risk behaviour, are sometimes used to justify infidelity and contribute to unhappiness.^3,4,34^

Sexual history taking is not a luxury but a necessity in the management of hypertension, dyslipidaemia, diabetes and cardiovascular diseases. In gaining a better understanding of the decision to take a sexual history, it is prudent to explore patients’ and doctors’ perspectives. To empower both doctors and patients to discuss sexual dysfunction to improve patient well-being and management, we need to deal with the perceived barriers in sexual history taking.

## Aim of the study

This research aimed to explore doctors’ and patients’ perspectives on sexual history taking during routine primary care consultations with patients at risk of sexual dysfunction in North West province, South Africa. This article will report how patients and doctors view talking about sexual matters and their perspectives on barriers to and facilitators of sexual history taking.

## Methods

### Study design

This study formed one of the six data sets that contributed to a broader grounded theory research^35^ that observed 151 video-recorded consultations of patients older than 18 years, who were at risk of sexual dysfunction because of their diagnosis of hypertension and diabetes and the medication they used. This article reported on the data set dealing with open-ended questions in the demographic questionnaire, as well as individual interaction and comments documented as field notes.

### Setting

Dr Kenneth Kaunda Health District, in North West province, was the selected research site. Mining and farming activities form the basis of socio-economic activities in the area. Approximately a quarter of the district’s population live in informal housing with poor to no infrastructure, such as piped water and sanitation. The Health District covers an area of 14 767 km^2^ and 28 doctors and numerous nurses provide healthcare to 707 479 individuals in 26 clinics and nine community health centres.^36^ Patients attending these clinics have no choice of the doctors they consult, and doctors, especially the interns and community service doctors, rotate through various clinics, which means patients often see a new doctor each time.

### Study population and sampling strategy

The broader methodology of this study was reported in another article.^37^ In short, there were three sampling strategies used: sampling of all the clinics with a doctor consulting at least once a week, followed by convenience sampling of all the doctors working at the sampled clinics and purposive selection of patients living with diabetes and hypertension sampled consecutively as they consulted. A total of 11 clinics and 21 doctors participated in this study. Numerous studies have shown that disclosure of sexual dysfunction varied between 14% and 20%.^38,39,40^ If the midpoint is 17%, the researcher needed a sample size of 151 consultations to observe sexual history taking events, based on a 95% confidence interval. The sample size calculation was performed on nQuery Advanced (Statistical Solutions Ltd, Cork, Ireland), Release 8.4.

### Data collection

A trained research assistant was tasked with obtaining consent from patients for the video recording of the consultation and the completion of the questionnaire following the consultation. Those patients who were too sick to participate and those who left directly after the consultation without completing the questionnaires were excluded from the study. The researcher was involved in taking doctors’ consent for the video recording of the consultation and completion of demographic questionnaires, and at the end of the day after completing all the recordings, consent for the interviews. Patients and doctors received notification that the content of the consultation would be analysed, but not about the sexual dysfunction aspect as it could sensitise them. Permission from the publisher was not granted for the translation of the sexual dysfunction questionnaires; therefore, the demographic questionnaires were also performed in English. The research assistant speaks five of the 11 national languages and assisted patients in their home language in the recruiting and consenting phase of the study. Most of the patients understood English and Afrikaans, but the research assistant translated different concepts and definitions in questionnaires to patients in Tswana, Sotho or Zulu when requested.

### Data analysis

Data were obtained from open-ended questions in the demographic questionnaire and comments were documented in field notes. Patients’ and doctors’ comments captured in the field notes were qualitatively analysed using MaxQDA 2018. After reviewing the qualitative data, open inductive coding developed concepts from raw textual data, with a focus on seeking to understand the barriers to and facilitators of sexual history taking in the consultation, as perceived by patients and doctors. Axial coding systematically interconnected the categories into broad categories. Themes emerged for patients and doctors from the selective coding, which will contribute to the broader theoretical theme. Constant comparison was performed between and within data sets and the broader study could conclude with a theoretical direction.^35^ A panel consisting of one family physician, a public health specialist and a general practitioner ensured agreement on the coding of the qualitative data.

### Ethical considerations

Ethical clearance to conduct this study was obtained from the Human Research Ethics Committee (Medical) of the University of the Witwatersrand (Number M160557).

## Results

A total of 21 doctors consulted 151 patient participants living with diabetes and hypertension in this study. There was no sexual history taken for sexual dysfunction except in five (3%) consultations, where basic history taking attempted to address other sexual health topics. These findings were reported in another article.^41^

### Participants’ demographic data

A total of 47 male (31%) and 104 female (69%) patients, as well as 21 doctors (15 men and 6 women) participated in this study. The biggest proportion of patients (60%) was Setswana-speaking; 55 (36%) were married and 32 (28%) were single. The median ages for patients were 49 and 55 years (men and women, respectively), with the youngest female participant 19 years old and the youngest man 26 years old. The female doctors (26–34 years) were mostly younger than their male colleagues (25–67 years) and both were either married or single. Four (19%) doctors shared the home language of the area, namely Setswana. The doctors and the patients differed in literacy levels, with 61 (40%) patients having only completed primary school education.

The male doctors had on average 21 years of work experience (median 16 years). Female doctors had less work experience, with a mean of four years and a median of three years. The participants were familiar with nursing staff and clinic routine as 141 (93%) patients had consulted before at these clinics.

### Patients’ perspectives on sexual history taking

**FIGURE 1:**
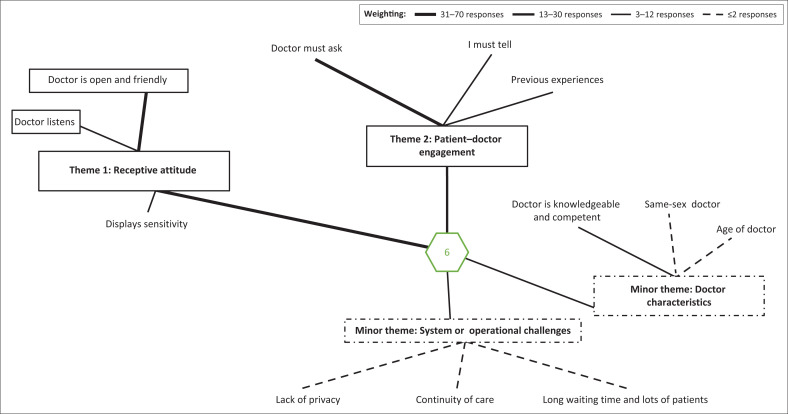
Patient factors influencing willingness to discuss sexual challenges.

Analysis of questions dealing with perceived barriers to and facilitators of sexual history taking revealed that patient participants had strong opinions on what would ease discussion of sexual matters with doctors (see Figure 1).

Two major themes reflect patients’ perceptions influencing disclosure of sexual challenges, namely a receptive attitude and patient–doctor engagement (Figure 1). Receptive attitude, which is defined as the ability or skill to accept new information and be responsive to other people or ideas, included subthemes of openness and friendliness, listening and sensitivity or patient-centredness.

Openness or friendliness was the primary subtheme and referred to a person who is pleasant and easy to talk to and share ideas with (Figure 1):

‘I will need the doctor to show kindness’. (Patient 70, 49-year-old male)

‘The doctor must show humanity and give attention’. (Patient 74, 93-year-old female)

‘The doctors must be open and friendly to me and then I can trust them with my sensitive issues’. (Patient 81, 64-year-old male)

The need for the doctor to listen was important for patient–doctor engagement. Listening denoted hearing, interpreting and responding to what the patient was saying – it usually leads to more questions, appreciation, empathy, comprehension or critical thinking actively and effectively:

‘The doctor does not address my fears – why discuss anything else? … No one goes out of [*their*] way to help’. (Patient 138, 67-year-old female)

‘Doctors listening to us, spend time to listen to us’. (Patient 94, 81-year-old female)

‘[*I will not discuss sexual matters*] If he does not have many questions and I have to ask more than he does’. (Patient 10, 46-year-old female)

Receptiveness (Figure 1) included a sensitivity, suggesting that patient care is respectful of, and responsive to, the patient’s individual experiences, preferences, needs, culture and values:

‘If he asked, I would tell him my husband cannot do it anymore and doing it by hand takes too long … I am diabetic and get infections down there’. (Patient 145, 65-year-old female)

‘I cannot tell about traditional stuff like going to the mountain as they will not understand’. (Patient 91, 26-year-old female)

‘When I see he or she is sensitive to my problem and is attentive to what I say and feel’. (Patient 4, 36-year-old female)

The second major theme was patient–doctor engagement, which represented the ability and willingness to choose to participate actively in the clinical encounter in order to achieve optimal clinical outcomes. Subthemes included perceptions of who must initiate the discussion of sexual problems and previous experiences (Figure 1):

‘The doctor must ask, as I will have knowledge of what I have and the problems I am accounting’. (Patient 30, 50-year-old male)

‘The doctor must ask me’. (Patient 2, 37-year-old male)

‘The doctor we have now can ask anything and I will tell him’. (Patient 43, 48-year-old female)

‘I must ask, it will help me to know how to protect my loved ones’. (Patient 14, 24-year-old female)

The subtheme previous experiences suggested patients’ attempts to discuss sexual dysfunction in previous consultations:

‘For [*the*] past 5 years [*I had*] no erection. Long ago I asked the doctor about it, but the doctor said I am too old for sex’. (Patient 85, 62-year-old male)

‘Still dreaming about it. Used to help myself after the wife died, but now (*I am*) too sick’. (Patient 105, 74-year-old male)

Patient 19 said:

‘What does it help you talk about it, they just worry about you drinking your medicine – if 4–5 [referring to penis] is weak, they do not worry. They do not hear us’. (36-year-old male)

Two minor themes for patients that emerged were doctor characteristics and system or operational challenges (Figure 1). Doctor characteristics referred to an attribute or quality ascribed to the doctor and system or operational challenges referred to the day-to-day management, organisation or routine at the clinics.

Doctor characteristics linked the discussion of sexual challenges with the level of knowledge and competence, gender and age of the doctor. The doctor had to be knowledgeable and competent and well informed:

‘Nothing [*must stop me to disclose*], because my doctor is the one who can give a right light to my sexual problems’. (Patient 125, 64-year-old female)

‘When the doctor shows me his capabilities … I will tell them my problem’. (Patient 54, 53-year-old male)

The gender of the doctor played a role. It referred to similar gender of a patient and doctor or the role gender plays in the initiation of the discussion:

‘If the doctor is a female’. (Patient 98, 47-year-old female)

Age of the doctor suggested that the age gap between patient and doctor must not be too big.

‘[*I will tell*] when the doctor is matured enough, not the children we see’. (Patient 82, 51-year-old female)

The system or operational theme dealt with day-to-day management, organisation or routine at the clinics and had three subthemes, namely lack of privacy, workload and related waiting times and continuity of care.

Lack of privacy referred to situations in which the doctor and the patient were interrupted or overheard by other people and included an area where the patient could be examined without the risk of being seen by another person:

‘Privacy. There should be no nurse in the consultation room’. (Patient 123, 55-year-old male)

Waiting times and workload referred to the number of patients for consultation or the time the doctor spends with each patient that causes longer waiting times:

‘When the doctor has time. We wait so long … they have too many patients’. (Patient 150, 70-year-old male)

Continuity of care: Patients wanted the same dedicated doctor they could consult regularly:

‘[*I*]f the doctor makes me feel comfortable after a long relationship with the same doctor’. (Patient 110, 46-year-old female)

### Doctors’ perspectives on sexual history taking

**FIGURE 2:**
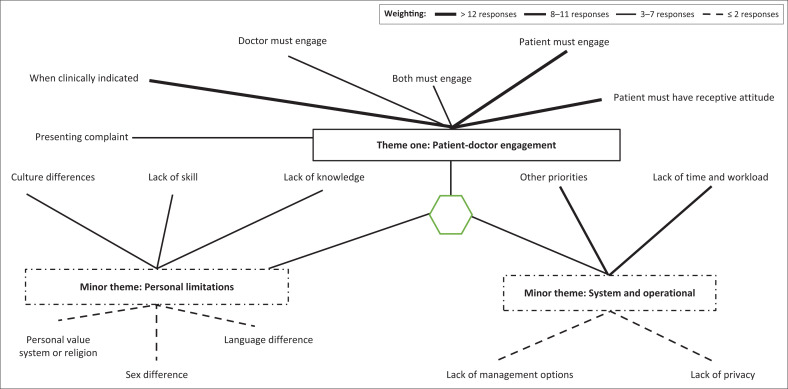
Doctors’ responses on facilitators and barriers of sexual history taking.

One major theme and two minor themes emerged from the doctors’ perceptions (Figure 2). The main theme was patient–doctor engagement, which reflected on the ability and willingness to choose to participate actively in the clinical encounter to have optimal clinical outcomes. Subthemes were who must initiate the discussion on sexual challenges, the symptom as presenting complaint, exploring sexual dysfunction if it is clinically indicated and the patient’s receptive attitude that will facilitate such a discussion.

Initiation of the discussion of sexual challenges appeared to be mainly the responsibility of the patient:

‘I often ask about HIV [*human immunodeficiency virus*] and STI [*sexually transmitted infection*], but not sexual dysfunction. I assume if it is a problem, they will raise it’. (Doctor 04, 25-year-old, male doctor)

‘So, who do they think must help them? That is not something one … uhm … I will address if somebody does not talk to me about it’. (Doctor 11, 28-year-old, female doctor)

‘Hmmmm … I don’t know. [*Doctor laughs*] Wait! They expect us to ask that?’ (Doctor 07, 47-year-old doctor)

Addressing sexual dysfunction is only when it is the presenting complaint, for example, when the patient presents at the doctor complaining of sexual challenges and seeks help for it:

‘If it is a problem, they will come to me and say this is the problem today. Till they seek help for it, it is not a problem’. (Doctor 20, 55-year-old male doctor)

Doctors indicated that they would explore sexual dysfunction if clinically indicated. Clinical indication referred to when the sexual challenge is involving or related to clinical treatment:

Doctor 17: ‘Look, we have so much to do. I cannot ask everything. When it is indicated I will ask’. (25-year-old male doctor)

Researcher: ‘Is it indicated in diabetic and hypertensive patients?’

Doctor 17: ‘In very few of them – usually after they have been sick for years’.

‘… if indicated but we see not a lot of them. I know the patient and read the file. It will be indicated’. (Doctor 14, 39-year-old male doctor)

Doctors believe that patients must indicate a receptive attitude to discuss sexual challenges. They must demonstrate the ability or skill to accept new information and be responsive to other people, their needs or ideas:

‘Patients must also be open and willing to discuss sensitive topics’. (Doctor 14, 39-year-old male doctor)

One minor theme was system or operational challenges, which signified the day-to-day management, organisation or routine at the clinics. The lack of time and workload, as well as other priorities, were prominent subthemes and often interlinked.

Doctors mentioned that they had other priorities to consider. Other priorities described disease management as complex and multifaceted; it also referred to control targets, side effects of medication and adherence. Linked to priorities was the lack of time and the number of patients for consultation, leading to insufficient time for the doctor to dedicate to one patient.

Reflecting on a patient in a previous consultation, Doctor 01–(37–year-old male), shared:

‘… after I spoke to him for such a long time, I think because he realised, okay, he can talk to me, then he opened up and he said, he has a girlfriend and he’s not able to perform … so I said, okay, no problem, we’ll sort it out, but obviously the hypertension is life threatening, this on the side is not, so it is not a priority’.

Another doctor stated:

‘We have so much to do and to check, and there is this long queue of patients waiting, even if I did think about it, it is not a priority’. (Doctor 10, 26-year-old male doctor)

The lack of privacy and a shortage of management options, in terms of resources for treating sexual dysfunction, were subthemes of the system and operational challenge minor theme. Lack of privacy meant consultation interruptions or other people overhearing. It included areas where patients can undergo examinations without the risk of others seeing:

‘I cannot talk about these things with the nurses in and out of the room – the patients are their neighbours, friends …’ (Doctor 12, 31-year-old female doctor)

Lack of management options where the healthcare system does not have medication or other resources to manage sexual dysfunction:

‘Howa! [a *South African expression of surprise or indignation*] You want me to talk about things like ED [*erectile dysfunction*] and then I cannot give something to help? If you talk about it, patients want something to make it better. We have nothing’. (Doctor 20, 55-year-old male doctor)

The second minor theme was personal limitations. This depicted personal areas that doctors see as lacking in themselves, which can be addressed with professional development. Six subthemes emerged.

Lack of skill (no skill to initiate discussions on sexual matters):

‘I do not know how to start the conversation’. (Doctor 06, 40-year-old male doctor)

The lack of knowledge (about sexual dysfunction and its management):

‘Well, it depends if it’s like … if it’s an erectile dysfunction, then that I have some idea but to be honest, anything other than that, not really … not really …’ (Doctor 01, 37 years old male doctor)

Language differences (language can differ in vocabulary, dialect and have different social connotations attached to words):

‘Patients talk in codes when they referred to sexual organs and sexual activities. I also cannot speak Tswana, so I cannot speak to the heart of the patient’. (Doctor 25, 26-year-old female doctor)

Culture and gender differences (culture differences and its impact on discussion of matters of sensitive nature and gender differences or similarities between a patient and doctor or the role gender plays in the initiation of the discussion):

‘In my culture you do not go around and ask a lady about sexual issues. It is not done. What in any case if they think it is sexual harassment?’ (Doctor 03, 35-year-old male doctor)

‘It is easier if it is a man’. (Doctor 14, 39-year-old male doctor)

Personal values and religion (it inhibits discussing sexual matters with some groups of people):

‘It is against my values and religion to talk about sex with patients who are not married’. (Doctor 18, 55-year-old male doctor)

**FIGURE 3:**
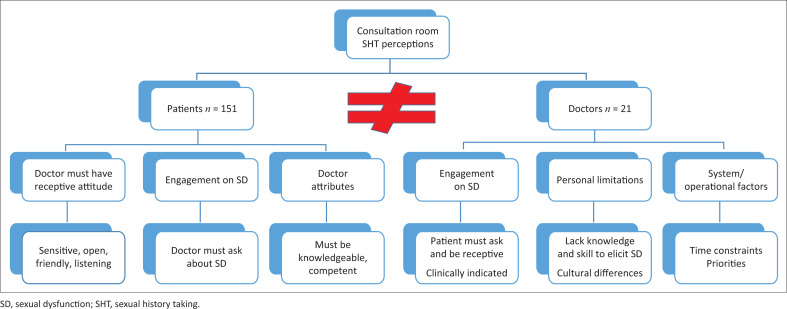
Key findings on barriers to and facilitators of sexual history taking.

The core finding of this study was the total disconnect between patients and doctors (Figure 3).

The patients’ and doctors’ expectations differed substantially. Patients wanted a friendly doctor who could listen to them. They trusted the doctors to share their skills and knowledge and because he is perceived to know more and thus to ask questions about their sexual challenges. By contrast, the doctors thought that despite being comfortable discussing sexual dysfunction, they have too much to do in a short period of time focusing on treatment priorities and if the patient has sexual dysfunction, the patient will and must tell them.

## Discussion

When exploring doctors’ and patients’ perspectives on sexual history taking during chronic primary health care consultations with patients at risk of sexual dysfunction in North West province, South Africa, it was clear that there is a critical disconnect between the two regarding their perspectives on and expectations of the discussion of sexual dysfunction during a consultation. Both groups shared the perception that the patient–doctor engagement is essential for such a discussion to take place; however, they perceived it differently. Doctors expected patients to present with sexual challenges, otherwise they would not ask. Conversely, patients expected doctors to ask about sexual challenges, otherwise they would not speak about them. It is noteworthy that receptiveness (openness, friendliness) emerged as important for both groups (as a major theme for patients and a subtheme for doctors), yet the extent to which this actually occurred in consultations was clearly part of the disconnect because the experience of the patients was that doctors failed to listen to them; this was supported by patients sharing negative experiences of what happened when they raised sexual challenges with their doctors. Listening is a core skill in communication and determines the quality of the consultation.^42^ From patients’ accounts they did not feel the doctors really listened to them, alternatively they were not heard. The patients experienced the patient–doctor engagement as more of a one-sided checklist exercise than a meaningful interaction. When the doctor does not apply the ability or skill to accept new information and perceived as unresponsive to the patient, their needs or ideas, the patients do not reciprocate; doctors then perceive the patient as non-receptive, which becomes a barrier in the interaction process.

Although both groups mentioned receptiveness as a factor for interaction, the presentation of the sexual challenge, as well as the knowledge and the skill of the doctor, played a role in initiating the discussion. Not introducing the topic of sexual dysfunction has severe implications for the patient’s interpersonal relationship and overall well-being.^10,43^ Patients had an expectation that the doctor knew more and would thus ask them. A study on help-seeking behaviour and sexual dysfunction conducted in Ghana found that 31% of 407 women did not know sexual dysfunction was a medical condition, whilst 29% thought it was normal.^44^ If a doctor does not make the link for the patient and diagnose sexual dysfunction, there will be no discussion during the clinical encounter and the patient will not receive help or gain a better understanding about the problem. By contrast, doctors expected patients to raise sexual dysfunction, either as a presenting complaint or during the consultation. Doctors left an opening, saying that if it were clinically indicated, they would discuss it. However, they consulted numerous patients living with diabetes and hypertension, the disease and treatment are known to cause sexual dysfunction, yet this was not enough of a clinical indication to discuss sexual challenges. It is thus difficult to understand what might be considered an appropriate clinical indication. This was not explored, but it seems there was an expectation that the ‘clinically indicated’ was seen to be a sexual challenge raised as part of a presenting complaint. It seems doctors are trained to use algorithms in learning material that mostly focus on initiating the sexual history when the patient presents with a sexual challenge or with a sexually transmitted infections and human immunodeficiency virus (HIV).^2,45,46^ Researchers also agree that a structured approach such as the Permission, Limited Information to dispel myths, Specific Suggestions directly related to the particular problem and Intensive treatment (PLISSIT) model and communication skills are crucial tools for the doctor to introduce sexual history.^22,47,48^ In this study, the patients’ and doctors’ disconnect seemed to be the biggest barrier in history taking for sexual dysfunction.

Patients and doctors also shared an understanding that what happened in the consultation, including sexual history taking, was influenced by the characteristics of the two parties involved, as well as system or operational challenges of the context in which this consultation occurred. Age, gender, language and cultural differences were common characteristics identified by both groups as barriers to the discussion of sexual dysfunction. Surprisingly, the participants revealed time constraints as a barrier, but did not attribute the same importance to gender, culture and age as barriers to sexual history taking that other studies have found.^2,17,22,49,50^ This could be contributed to mindfulness because of the research methodology of recording actual consultations that heightened the actual awareness of being in the moment of the consultation where other factors were more prominent than age, culture or gender. In South Africa, one cannot ignore diversity. Although not mentioned by the participants in this study, it is known that gender roles, health literacy and the inability to express oneself in a second language, which are part of the diversity issue, can compromise the patient–doctor engagement too.^51,52^

The doctors raised lack of knowledge as a barrier, which also contributed to the perception of a lack of management options. Whilst it is true that the public healthcare sector in South Africa does not have medication to treat sexual dysfunction at primary care level, it is a misconception that only medication is needed to manage it. It is known that psychosocial intervention for sexual dysfunction can also change well-being of patients, more so for female patients.^53^

Both patients and doctors mentioned long waiting times, privacy and workload. Although not considerable in terms of the number of responses in this study, time constraints have been indicated in other studies to be barriers to sexual history taking.^17,50^ Time constraints and workload as a minor theme were another surprise. It is common knowledge that the South African public health sector is under pressure. Besides the number of doctors, South Africa is plagued with the quadruple burden of disease (communicable diseases [HIV, tuberculosis]; non-communicable diseases; maternal and child health; and violence and injury), all of which lead to extended consultations. Gude et al.^54^ showed that a few more minutes in a consultation actively engaging with a patient, makes a difference in the patient–doctor relationship and consultation outcomes. A point of concern, but also seen in the context of time constraints and workload, is the perception that doctors have other priorities than considering sexual well-being during the consultation. Doctor 01, on reflecting on a previous patient encounter, decided with confidence that the patient’s hypertension was more important than the concerns the patient expressed, despite the patient raising the concern. Kingsburg^2^ also suggested that patients feared their complaints would be dismissed despite verbalising sexually challenges. It seems in this study, the doctor set the agenda for the consultation, thus negating the patient’s needs, with no negotiation on management. Could training change patients’ and doctors’ perceptions?

Continuous evaluation and training form the core of professionalism. Numerous studies have suggested that training on sexual history taking changes the practice for doctors.^12,18,55,56^ As it is an interactive process between doctors and patients, we can start to improve awareness of patients. Pamphlets or posters could increase patients’ awareness of their right to discuss sexual dysfunction with their doctors. It will however not change the doctors’ interaction if they do not listen to patients or continue to determine the priorities in the consultation. The question is also if it is ethical to make the patient responsible if the doctor has a duty to address disease, its comorbidities and the possible side effects of medication. To improve the quality of the clinical outcome, we need to make doctors culturally competent and consequently improve communication. This means an awareness of the linguistic and cultural diversity that goes beyond race and ethnicity, how it impacts the help-seeking behaviour and the understanding of disease and expectations of the clinical encounter.^52,57^ Patient–doctor communication on sensitive matters must also improve significantly.^52^ Furthermore, doctor’s attitudes regarding their role to make decisions on behalf of a patient must change; they need to reflect on their commitment to patients and their care and ensure continuity of care to build trust and collaboration.^58^ A patient-centred approach, where both the doctor and the patient participate in the consultation and negotiate priorities and management thereof, will improve the chances for a patient to disclose sexual dysfunction and get appropriate help. As a last resort, continuous medical education can improve doctors’ knowledge regarding the diagnosis of sexual dysfunction and its different treatment modalities.

### Limitations

It was not possible at the time of data collection to have in-depth interviews with patients and interaction with participants was limited to the questionnaire completion and spontaneous comments documented as field notes. Sexual dysfunction is multifaceted and so is the disclosure thereof. Help-seeking behaviour and health literacy would be valuable information to add to these findings. The results of the study apply to the context of the research.

## Conclusion

This study concluded that a disconnect between patients and doctors caused by the doctor’s perceived clinical priorities and expectation that a patient must raise the topic inhibited sexual history taking. Factors that could improve history taking were that the patient must either present with sexual dysfunction or tell the doctor. Overall, a lack of cultural competence and the absence of an inclusive patient-centred approach contributed to missed opportunity to screen for sexual challenges.
